# Inhibiting triggering receptor expressed on myeloid cells 1 signaling to ameliorate skin fibrosis

**DOI:** 10.1172/jci.insight.176319

**Published:** 2024-12-06

**Authors:** Swarna Bale, Priyanka Verma, Bharath Yalavarthi, Matija Bajželj, Syed A.M. Hasan, Jenna N. Silverman, Katherine Broderick, Kris A. Shah, Timothy Hamill, Dinesh Khanna, Alexander B. Sigalov, Swati Bhattacharyya, John Varga

**Affiliations:** 1 Michigan Scleroderma Program, Division of Rheumatology, Department of Internal Medicine, University of Michigan, Ann Arbor, Michigan, USA.; 2 SignaBlok Inc., Shrewsbury, Massachusetts, USA.

**Keywords:** Autoimmunity, Infectious disease, Autoimmune diseases, Rheumatology

## Abstract

Systemic sclerosis (SSc) is characterized by immune system failure, vascular insult, autoimmunity, and tissue fibrosis. TGF-β is a crucial mediator of persistent myofibroblast activation and aberrant extracellular matrix production in SSc. The factors responsible for this are unknown. By amplifying pattern recognition receptor signaling, triggering receptor expressed on myeloid cells 1 (TREM-1) is implicated in multiple inflammatory conditions. In this study, we used potentially novel ligand-independent TREM-1 inhibitors in preclinical models of fibrosis and explanted SSc skin fibroblasts in order to investigate the pathogenic role of TREM-1 in SSc. Selective pharmacological TREM-1 blockade prevented and reversed skin fibrosis induced by bleomycin in mice and mitigated constitutive collagen synthesis and myofibroblast features in SSc fibroblasts in vitro. Our results implicate aberrantly activated TREM-1 signaling in SSc pathogenesis, identify a unique approach to TREM-1 blockade, and suggest a potential therapeutic benefit for TREM-1 inhibition.

## Abstract 2

We demonstrated the antifibrotic potential of TREM-1 blockade in systemic sclerosis (SSc) by animal model of fibrosis and SSc.

## Graphical abstract

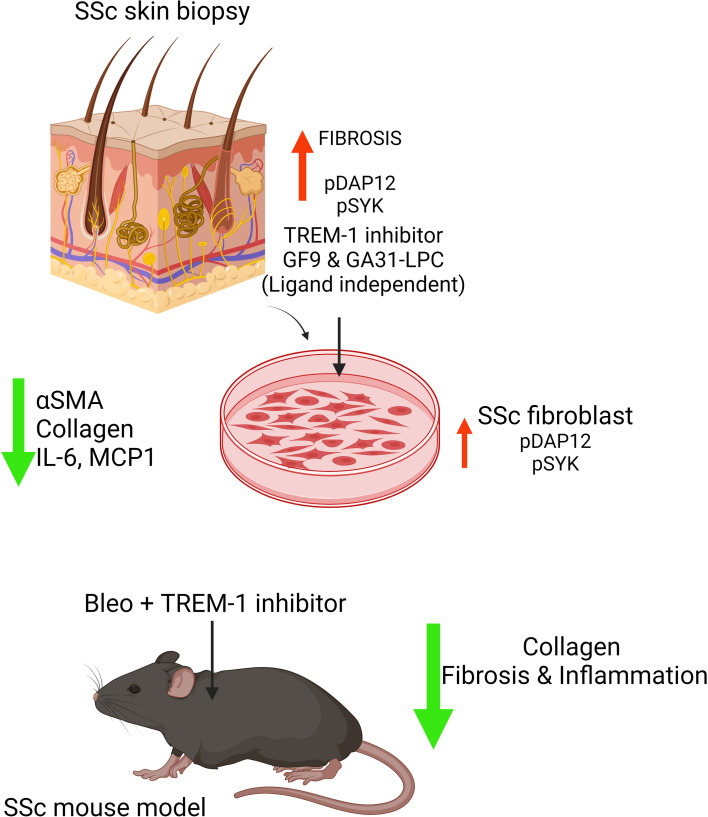

## Introduction

Fibrosis that affects multiple organs is the hallmark sign of systemic sclerosis (SSc) and lacks effective therapy (1, 2). Fibrosis in SSc is associated with high mortality (3–5). Numerous intracellular signaling pathways are implicated as drivers of SSc fibrosis, but the nature of their continuing dysregulation in pathological inflammation and fibrosis remains poorly understood. Recent studies have uncovered an essential role for innate immunity in the pathogenesis of SSc (6). As a component of the innate immune system, the cellular receptor triggering receptor expressed on myeloid cells 1 (TREM-1) is expressed on neutrophils, monocytes, macrophages, and endothelial cells (7, 8). Endogenous ligands for TREM-1 include high-mobility group box 1 (HMGB1), heat shock protein 70 (Hsp70), peptidoglycan recognition protein 1 (PGLYRP), and extracellular cold-inducible RNA-binding protein (eCIRP1) (9). Activation of TREM-1 leads to phosphorylation of its signaling partner DNA-activating protein of 12 kDa (DAP12), which induces cytokine and chemokine production (10, 11). However, the expression, role, and mechanisms of TREM-1 signaling in fibrosis in SSc are currently unknown (9, 12).

Upregulation of TREM-1 on immune cells is implicated in acute inflammation (7, 13), while sustained TREM-1 activation plays a crucial role in sepsis, arthritis, and colitis (11, 13–16). Notably, TREM-1 amplifies signaling from cellular pattern recognition receptors, such as TLR4, thus amplifying the inflammatory response (17, 18). In previous studies, we established an essential role for several extracellular matrix proteins as damage-associated molecular patterns (DAMPs) that function as endogenous ligands for TLR4 to drive sustained fibroblast activation underlying fibrosis progression in SSc (19, 20). In light of TREM-1’s potential to interact with the TLR4 signaling pathways, we sought to explore the involvement of TREM-1 in SSc and the impact of TREM-1 inhibition in preclinical models of fibrosis.

There have been substantial efforts to develop therapeutic TREM-1 inhibitors. Currently available TREM-1 blockers, including inhibitory peptides LR12 and M3 (21–25), the small-molecule VJDT (26), and an anti–TREM-1 antibody (27), are ligand-dependent inhibitors that block TREM-1 binding to its ligand(s) (28). At least 5 different molecules have been reported as putative TREM-1 ligands (29). In the pathogenesis of TREM-1–linked inflammatory diseases, the expression of these molecules depends on disease pathogenesis, stage, and severity, which might affect the efficacy of ligand-dependent TREM-1 inhibitors. Importantly, despite promising results in disease models in small and large animals and safety in humans (30–35), the first clinical TREM-1 blocker, LR12 peptide (Nangibotide), failed in a recent phase IIb sepsis trial (25).

In the present study, we sought to characterize the involvement of TREM-1 in SSc. We used unique TREM-1 inhibitors to determine the impact of TREM-1 in preclinical disease models. These inhibitors were created based on the TREM-1 inhibitory peptide sequence GF9 (reviewed in ref. 28). The free peptide GF9 and GA31 peptide formulated in macrophage-targeted lipopeptide complexes (GA31-LPC) employ a ligand-independent mechanism to disrupt protein-protein interactions between TREM-1 and DAP12 at the membrane (28). Systemically administered GF9 functions as a “pan–TREM-1” blocker on all TREM1-expressing cells (neutrophils, monocytes, macrophages), while GA31-LPC blocks TREM-1 primarily on macrophages (28). Treatment with GF9 and GA31-LPC was previously shown to suppress systemic and local inflammation and ameliorate disease in animal models of rheumatoid arthritis, alcoholic liver disease, retinopathy, and cancer (36–39).

Here, we demonstrate that TREM-1 signaling was activated in SSc skin biopsies and its inhibition mitigated constitutive collagen synthesis and the myofibroblasts phenotype in explanted SSc fibroblasts. Furthermore, ligand-independent selective TREM-1 blockade prevented and reversed bleomycin-induced fibrosis in mice. Altogether, these results implicate aberrant TREM-1 signaling in SSc and provide a rationale for further exploring selective TREM-1 targeting as a distinct therapeutic strategy.

## Results

### Treatment with TREM-1 inhibitors at an early time point prevented bleomycin-induced responses.

**Figure 1:**
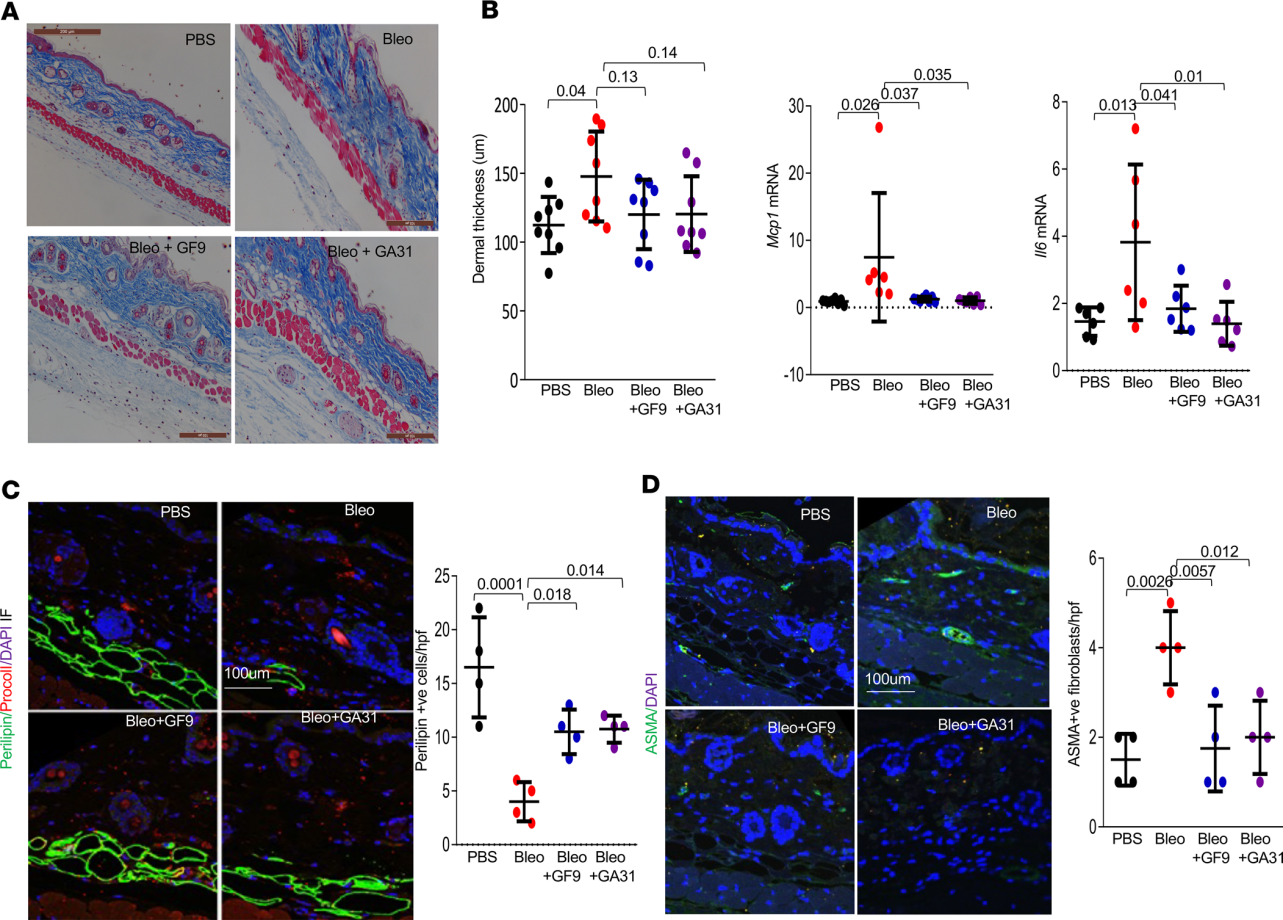
Pharmacological inhibition of TREM-1 prevents early loss of dermal adipose tissue. C57BL/6J mice received daily subcutaneous injections of PBS or bleomycin alone or together with GF9 and GA31-LPC or vehicle for 5 days. Mice were sacrificed on day 8, and skin was harvested for analysis. (**A**) Representative images of Trichrome staining. Scale bar: 100 μm. (**B**) Assessment of dermal thickness from **A** (8 determinations/hpf) and real-time quantitative PCR. Results were normalized with GAPDH and are shown as mean ± SD of triplicate determinations from 6 mice per group. One-way ANOVA followed by Šidák’s multiple comparison test. *P* values are as shown. (**C**) Immunolabeling using antibodies against perilipin (green), procollagen I (red), and DAPI (blue). Representative images and *P* values are shown. Scale bar: 100 μm. (**D**) Immunolabeling using antibodies against ASMA (green) and DAPI (blue). Quantification of ASMA-positive cells is shown (an average of 4 randomly selected regions from 4 mice/group). One-way ANOVA followed by Šidák’s multiple comparisons test. Scale bar: 100 μm. *P* values are shown.

In this study, we used the TREM-1 inhibitors GF9 and GA31-LPC for the first time to our knowledge. Eight-week-old female C57BL/6J mice were injected with bleomycin daily subcutaneously for 1 week (5 days/week), concurrent with vehicle or GF9 (25 mg/kg) or GA31-LPC (13 mg/kg) given by daily intraperitoneal injections (5 days/week). Mice were sacrificed on day 8. Masson’s trichrome staining of skin from mice treated with bleomycin, compared with vehicle-treated mice, showed decreased thickness of the cutaneous white adipose tissue (DWAT), associated with increased dermal thickness (Figure 1A). Dramatic bleomycin-induced loss of DWAT in mice was substantially attenuated by treatment with GF9 or GA31-LPC (Figure 1A). Loss of DWAT was further demonstrated using perilipin immunostaining (Figure 1C). Pharmacological TREM-1 blockade attenuated the increase in inflammatory cytokines, including *Mcp1* and *Il6* (Figure 1B). We observed no significant difference in dermal thickness and skin procollagen I levels with TREM-1 inhibitor treatment at this early time point (Figure 1C). We investigated the effect of GF9 and GA31-LPC on accumulation of ASMA-positive interstitial myofibroblasts and phosphorylated DAP12 (pDAP12; a marker for TREM1 activation) and CD45-positive leukocytes. Interestingly, 7 days of GF9 and GA31-LPC treatment decreased accumulation of ASMA-positive myofibroblasts (*P* = 0.0057 and *P* = 0.012) (Figure 1D) as well as pDAP12- and CD45-positive leukocytes (Supplemental Figure 1, A and B; supplemental material available online with this article; https://doi.org/10.1172/jci.insight.176319DS1).

### TREM-1 inhibition concomitant with bleomycin administration prevented skin fibrosis.

**Figure 2:**
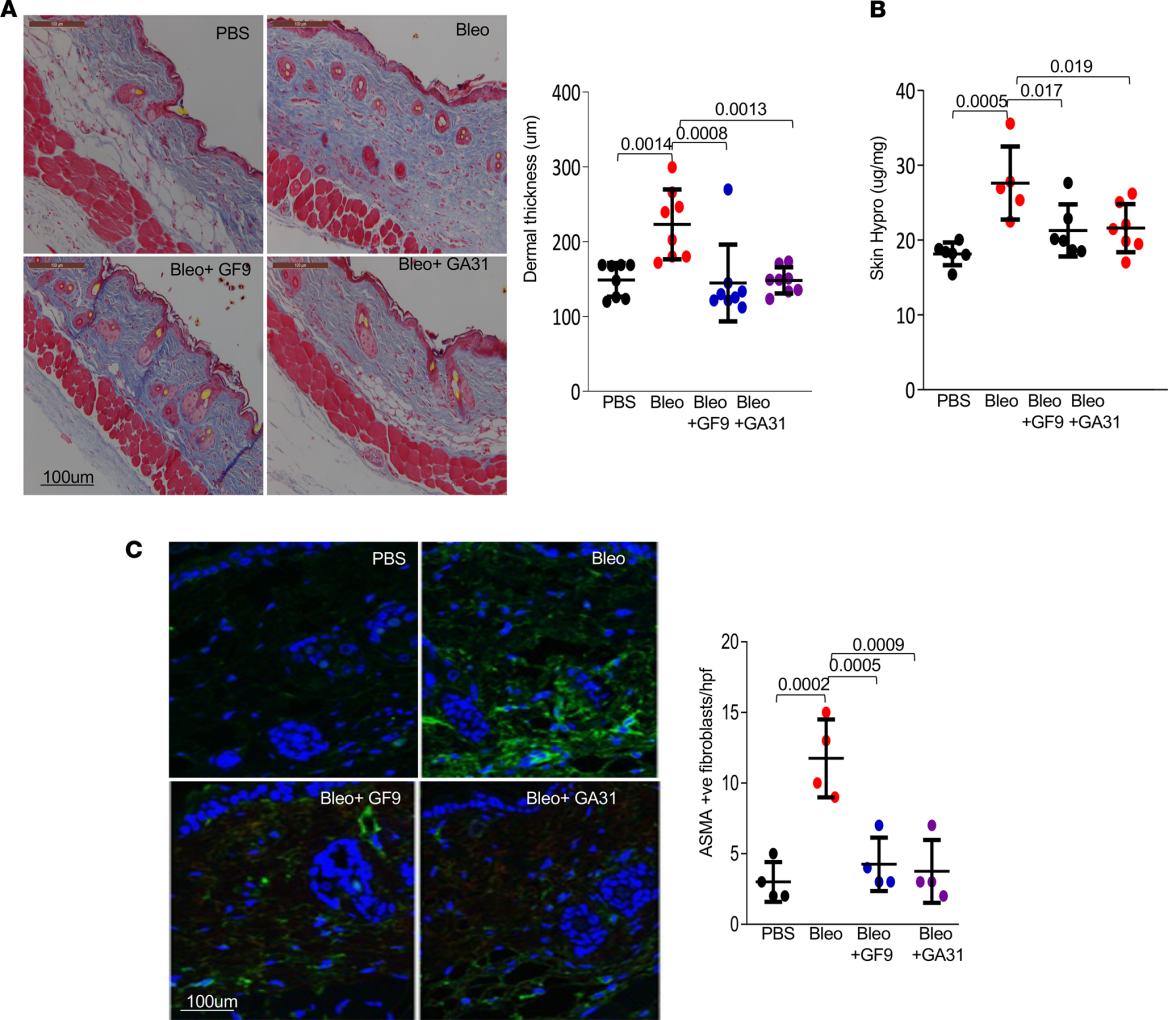
Inhibition of TREM-1 signaling by GA31-LPC treatment prevents skin fibrosis. C57BL/6J mice were treated for 2 weeks. They were sacrificed on day 22, and skin was harvested for analysis. (**A**) Representative images of Trichrome staining and assessment of dermal thickness. Scale bar: 100 μm. Results are shown as the mean ± SD of 8 determinations/hpf. One-way ANOVA followed by Šidák’s multiple comparisons test. *P* values are shown. (**B**) Skin hydroxyproline assays. Results are shown as mean ± SEM. *P* values are shown. (**C**) Immunolabeling using antibodies against ASMA (green) and DAPI (blue). Representative images are shown. Scale bar: 100 μm. ASMA-positive cells (an average of 4 randomly selected regions per group). One-way ANOVA followed by Šidák’s multiple comparisons test. *P* values are shown.

We next examined the effect of treatment with GF9 and GA31-LPC for 21 days. Treated mice showed no significant weight loss, and treatment was well tolerated with no behavioral changes or overt signs of toxicity. Bleomycin-treated mice showed increased dermal collagen accumulation, enhanced dermal thickness, and loss of DWAT, compared with PBS-treated mice (Figure 2A). Concurrent GF9 or GA31-LPC treatment resulted in reduced collagen deposition and dermal thickness and restored DWAT compared with bleomycin-treated mice (Figure 2, A and B). When compared with early time points, the skin at 22 days of bleomycin treatment demonstrated increased numbers of ASMA-positive interstitial myofibroblasts, which was attenuated in mice with TREM-1 inhibitors administered concomitantly with bleomycin (Figure 2C). Immunolabeling indicated a marked reduction in the numbers of CD45-positive leukocytes and CD11b-positive myeloid cells (pan myeloid marker) but no significant changes in T cell accumulation in the dermis from mice treated with TREM-1 inhibitors was observed (Supplemental Figures 2 and 4).

### Treatment with GF9 and GA31-LPC attenuated established skin fibrosis.

**Figure 3:**
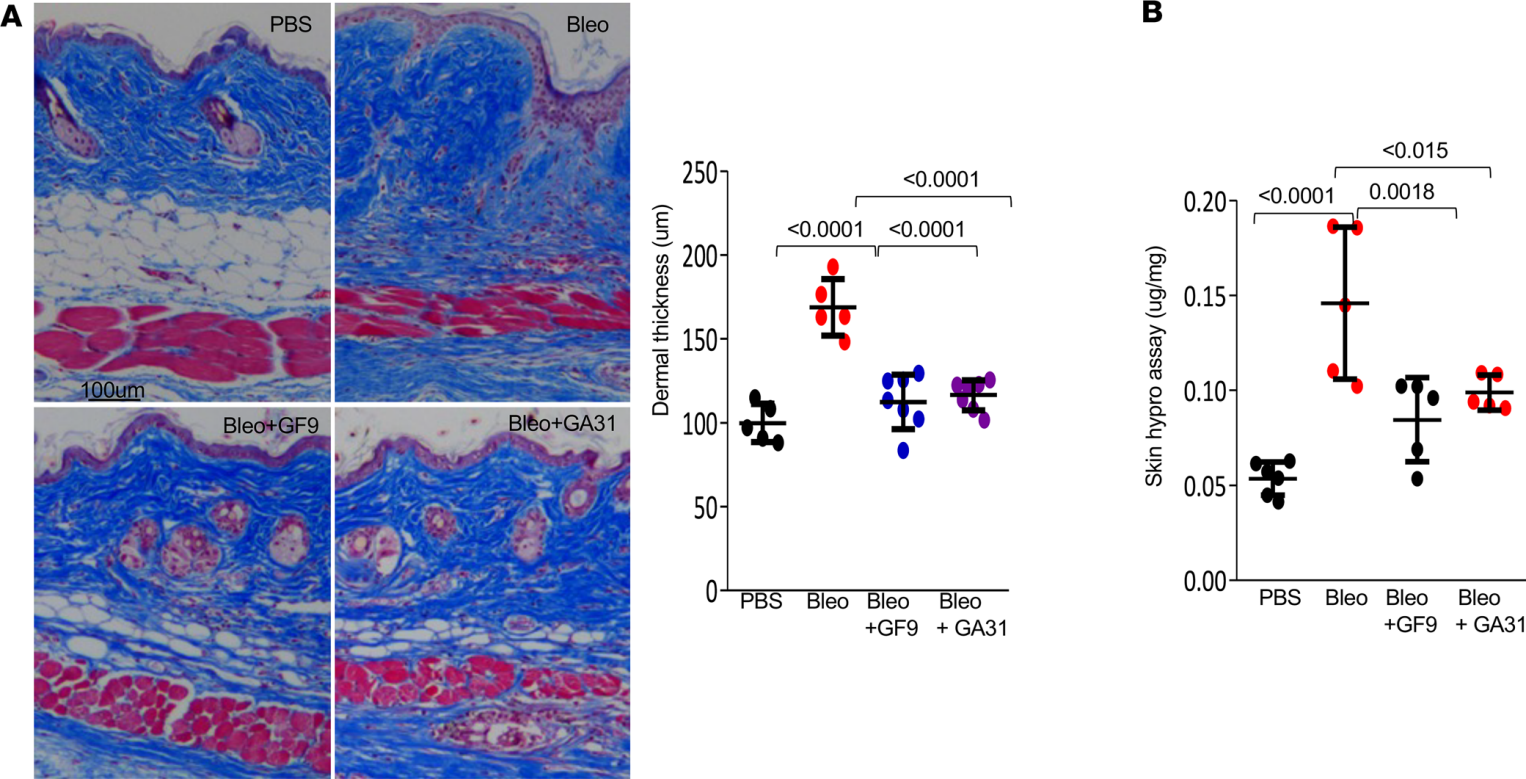
Inhibition of TREM-1 signaling by GF9 and GA31-LPC treatment mitigates established skin fibrosis. C57BL/6J mice were randomized to 4 treatment groups (*n* = 5–8 mice/group). They were euthanized on day 28, and skin was harvested. (**A**) Representative images of Trichrome staining and assessment of dermal thickness. Scale bar: 100 μm. Results are shown as the mean ± SD of 5–8 determinations/hpf. One-way ANOVA was followed by Šidák’s multiple comparisons test. *P* values are shown. (**B**) Hydroxyproline assays. One-way ANOVA followed by Šidák’s multiple comparisons test. *P* values are shown.

To evaluate the effect of TREM-1 inhibition in the therapeutic approach, we initiated TREM1 inhibitor treatment on day 15 following the start of bleomycin, when skin fibrosis is already established (40). Analysis of the lesional skin showed that treatment with GF9 or GA31-LPC attenuated the bleomycin-induced increase in dermal thickness and collagen accumulation (Figure 3), and restored DWAT. Chronic administration of GF9 and GA31-LPC in these experiments appeared to be well tolerated.

### TREM-1 signaling is activated in SSc skin biopsies.

**Figure 4:**
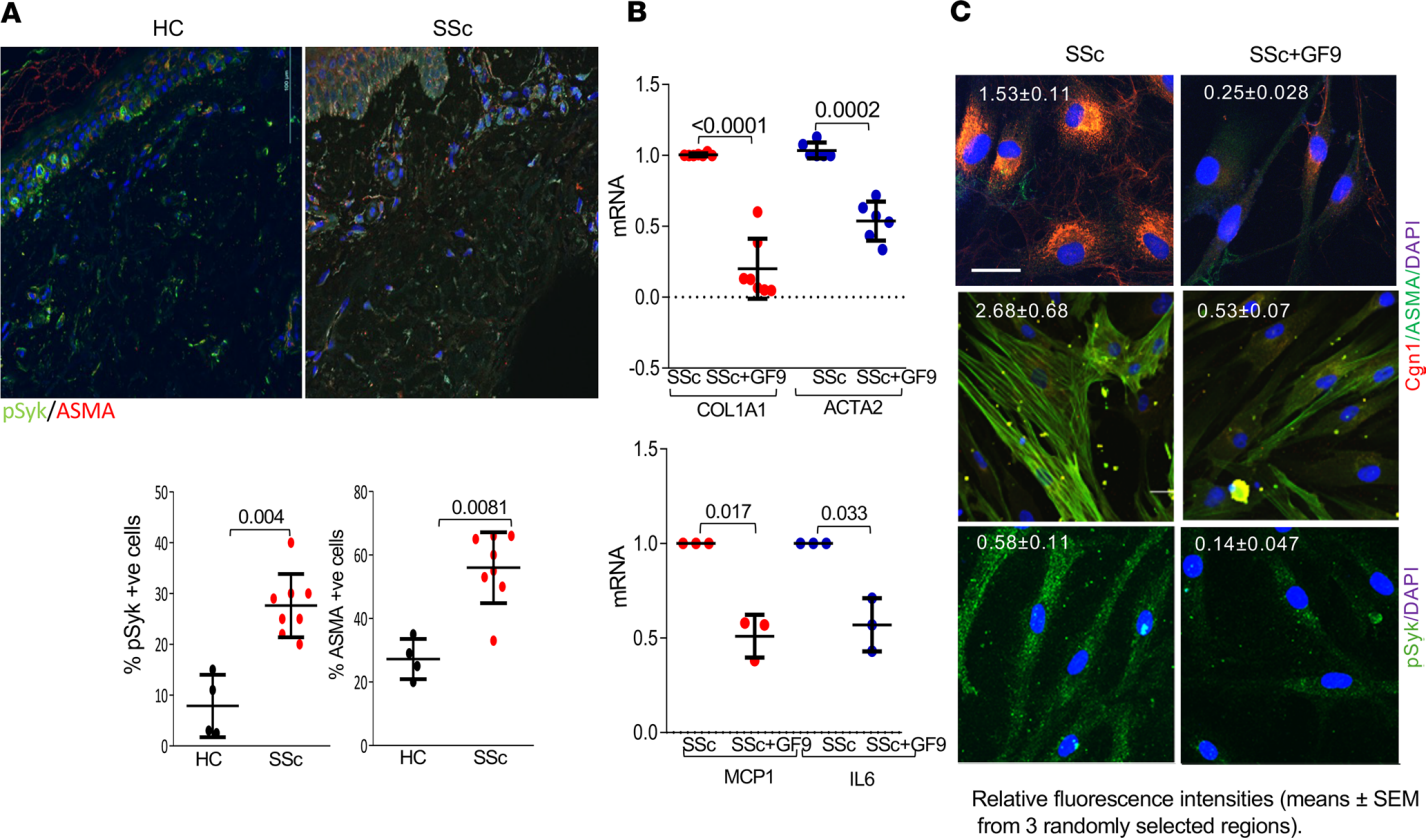
TREM-1 signaling is activated in SSc skin. (**A**) Skin biopsies from patients with SSc (*n* = 8) and individuals acting as healthy controls (*n* = 4) were immunolabeled with antibodies against pSyk or ASMA, and immunofluorescence was visualized by Nikon A1R laser scanning confocal microscope. The percentage of immuno-positive cells (mean percentages from 4 randomly selected regions) was quantified. Mann-Whitney *U* test. *P* values are shown. Original magnification, ×40. (**B**) Confluent SSc skin fibroblasts (*n* = 8, top; *n* = 3, bottom) were incubated with GF9 for 24 hours, and mRNA levels were quantitated by real-time quantitative PCR. Results were normalized with GAPDH and are shown as the mean ± SD of triplicate determinations from individual patients. Paired *t* test. *P* values are shown. (**C**) SSc fibroblasts (*n* = 6) were immunolabeled using antibodies against collagen I, ASMA, or pSyk. Scale bar: 100 μm. Representative images and relative fluorescence intensities (mean ± SEM from 3 randomly selected regions) are shown.

To characterize TREM-1 activity in SSc, we determined levels of phosphorylated Syk (pSyk), a marker of TREM-1 activation (41), in the skin. Immunolabeling of skin biopsies indicated significantly higher levels of pSyk (*P* = 0.004), accompanied by an increased number of ASMA-positive interstitial cells (*P* = 0.0081), in SSc skin biopsies compared with those from individuals acting as healthy controls (Figure 4A), while there was no significant difference in TREM-1 levels (data not shown). Furthermore, levels of the TREM-1 activation target DAP12 were elevated in explanted SSc fibroblasts compared with healthy skin fibroblasts (Supplemental Figure 3A). To investigate the effect of TREM-1 on explanted fibroblasts in vitro, confluent SSc skin fibroblasts were incubated in media with GF9 (10 μM) for 24 hours. GF9 treatment was associated with substantial downregulation of mRNA levels of COL1A1 and ACTA2 as well as inflammatory cytokines MCP1 and IL-6 (Figure 4B). Importantly, GF9 reduced the production of collagen I and cellular levels of ASMA, which was accompanied by a reduction in cellular pDAP12 and pSyk levels (Figure 4C and Supplemental Figure 3B). Together, these results indicate a potent antifibrotic effect of TREM-1 inhibition in explanted SSc fibroblasts.

## Discussion

Fibrosis in SSc affects the skin and multiple internal organs (42). The pathogenesis of SSc is still poorly understood, but emerging evidence implicates dysregulated innate immune signaling (43). Patients with SSc have limited therapeutic options, and unique therapeutic approaches are needed (44). TREM-1 is a widely expressed cellular receptor involved in innate immune signaling via the adaptor protein DAP12 (28). TREM-1 activation triggers phosphorylation and activation of the Src kinase Syk, resulting in Syk2 phosphorylation (41). The identity of TREM1 ligands remains incompletely established, and multiple putative endogenous ligands have been described (28). Blocking TREM-1 signaling has been explored as a potential approach to the treatment of inflammation-associated disorders, including sepsis, rheumatoid arthritis, and retinal neovascularization, as well as cancer (13, 36, 37, 39, 45).

Soluble TREM-1 is a glycoprotein derived primarily from the proteolytic cleavage of membrane TREM-1 and is a biomarker for TREM1 activation. Levels of sTREM1 were elevated in patients with diffuse cutaneous SSc and correlated with the severity of pulmonary fibrosis (30). These findings suggested that serum soluble TREM-1 could be a unique marker for disease severity (45). Furthermore, pDAP12, the marker for TREM-1 activation was elevated in skin biopsies from patients with early-stage SSc, as well as in the bleomycin-induced fibrosis model. Our current studies are the first to our knowledge to show that targeting TREM-1 using ligand-independent peptide inhibitors prevents and reverses pathological skin fibrosis in mice and represents a potential antifibrotic strategy for the treatment of fibrosis in patients with SSc.

The TREM-1 enhanced inflammatory response was observed in noninfectious disease models, including hemorrhagic shock, pancreatitis (acute inflammation), chronic inflammatory bowel diseases, and inflammatory arthritis (46–49). TREM-1–deficient mice displayed significantly reduced disease phenotype associated with reduced inflammatory infiltrates and diminished expression of proinflammatory cytokines, thus representing an attractive target for treating chronic inflammatory disorders (50). Such data are noteworthy in suggesting that TREM-1 also plays a regulatory role in influencing the disease outcome. Therefore, we pursued the inhibitors’ antifibrotic effect in a preclinical fibrosis model. Treatment with GF9 and GA31-LPC exerted potent antifibrotic effects in mice and mitigated the activated phenotype of SSc fibroblasts in vitro. Moreover, the inhibitors also showed antifibrotic effects on established skin fibrosis model. As expected, we have found downregulation of pDAP12 and levels of inflammatory and profibrotic cytokines (MCP-1 and IL-6), pan leukocyte marker CD45, and pan myeloid marker (CD11b) in mice. While the ligands of TREM-1 are still unknown, it has been shown that TREM-1 activation amplifies inflammation and synergizes with TLR signaling (51). We have shown previously that expression of TLR4 and its endogenous damage-associated ligands is elevated in patients with SSc (6, 20). Ligand-induced TLR4 activation in stromal cells elicits potent stimulation of fibrotic gene expression, myofibroblast transformation, and survival, thus contributing to fibrosis persistence and progression (19, 52). TREM-1 inhibitors prevented phosphorylation of cellular DAP12, an early event in both TLR4 and TREM-1 signaling. Therefore, blocking early events in fibrotic activation in stromal cells might represent a therapeutic approach to ameliorate fibrosis and merits further investigation.

## Methods

### Sex as a biological variable.

We used female mice (8–12 weeks old) in this study because they have better fibrotic responses in the subcutaneous bleomycin model compared with male mice and because SSc has a bias for female sex.

### Cell culture and reagents.

The synthesis of the 9- and 31-mer TREM-1 inhibitory peptides, GFLSKSLVF (human TREM-1213-221, GF9) and GFLSKSLVFPLGEEM(O)RDRARAHVDALRTHLA (GA31), was described previously (39, 41, 53).

Primary cultures of fibroblasts were established by explantations from skin biopsies from patients with SSc. Low-passage fibroblasts grown in monolayers in plastic dishes were studied as previously described (54). All SSc fibroblasts are derived from patients with early stage (<3 years from first non-Raynaud disease manifestation) disease. Clinical characteristics of participants in the study are listed in Table 1. All SSc skin biopsies were recruited from patients with diffuse cutaneous SSc. Control skin biopsies were recruited from healthy individuals.

### Model of dermal fibrosis.

Eight- to 12-week-old female C57BL/6J mice (The Jackson Laboratory) received subcutaneous injections of bleomycin (10 mg/kg/d) or PBS daily for 10 days (5 days/week). Daily intraperitoneal injections of GF9 (25 mg/kg) and GA31-LPC (13 mg/kg) were started concurrently with bleomycin, and mice were sacrificed on day 8 or day 22. Another group of mice received GF9 and GA31-LPC injections starting at day 15 of bleomycin treatment and continuing until sacrifice at day 28. A third group of mice received PBS, and a fourth received bleomycin alone. Tissue collagen content was determined using Colorimetric Assay Kits (Biovision).

Paraffin-embedded tissue sections (4 μm) were stained with Trichrome and analyzed as described previously (54). Skin collagen content was determined using hydroxyproline assays (Colorimetric Assay Kits, Biovision). Total RNA isolated from mouse skin was reverse transcribed to cDNA using Supermix and analyzed by real-time qPCR (Applied Biosystems) on an Applied Biosystems 7500 Prism Sequence Detection System as described previously (4, 54).

### Isolation and analysis of RNA from SSc skin fibroblasts.

At the end of the experiments, total RNA was isolated from SSc fibroblasts and reverse-transcribed to cDNA using Supermix (cDNA Synthesis Supermix; Quanta Biosciences) as described previously (55). Amplification products (20 ng) were amplified using SYBR Green PCR Master Mix (Applied Biosystems) on an Applied Biosystems 7500 Prism Sequence Detection System. Primer sequences are listed in Table 2. Data were normalized to GAPDH RNA, and the fold change in samples was calculated (55).

### Immunofluorescence confocal microscopy using SSc skin fibroblasts and skin biopsies.

SSc fibroblasts seeded on 8-well Lab-Tek II chamber glass slides (Nalgene Nunc International) were incubated in serum-free DMEM with or without GF9 (10 μM) for 24 hours. Cells were then fixed, permeabilized, and incubated with antibodies against ASMA (Sigma-Aldrich, 1:500, A5228), type I collagen (Southern Biotechnology, 1:100, 1310-01) and pSyk (CST 2710S), followed by Alexa Fluor–labeled secondary antibodies (Invitrogen) as described previously (55). For immunofluorescence, paraffin-embedded skin sections were incubated with antibodies against ASMA (Sigma-Aldrich, 1:100, A5228), pSyk (CST 2710S, 1:100), pDAP12 (ab314891, 1:100), CD45 (14-0451-82, 1:100), CD3(sc-20047; 1:100), TREM-1 (Invitrogen PA5-47090, 1:100), anti-CD11b antibody (Abcam AB133357, 1:100), procollagen I (Sigma-Aldrich, MAB1912 1:200), or perilipin (Abcam; ab61682) followed by appropriate secondary antibodies.

Nuclei were detected using DAPI. Slides were mounted, and immunofluorescence was evaluated in a blinded manner under a Nikon A1R laser scanning confocal microscope. Negative controls stained without primary antibodies were used to confirm immunostaining specificity.

### Statistics.

We used the Mann-Whitney *U* and Student’s *t* test (2 tailed) to compare 2 groups, with a *P* value correction for multiple comparisons. We presented the data as mean ± SD unless otherwise indicated. We examined the differences among groups for statistical significance using 1-way ANOVA followed by Šidák’s correction. A *P* value less than 0.05 was considered significant. We analyzed the data using GraphPad Prism (GraphPad Software version 8).

### Study approval.

Biopsies were performed with written informed consent, as per protocols approved by the IRB for Human Studies at Northwestern University and the University of Michigan (00186936). Animal experiments were performed according to protocols approved by Northwestern University and the University of Michigan and in compliance with the University of Michigan’s Animal Care and Use Committee guidelines (PRO00011706).

### Data availability.

All the raw and processed data are stored at the University of Michigan and are available upon request. Values for all data points in graphs are reported in the Supporting Data Values file.

## Author contributions

S Bhattacharyya, JV, and ABS conceptualized the study. S Bhattacharyya wrote the original draft of the manuscript, and JV and ABS edited it. S Bale, BY, and SAMH performed mouse experiments and analysis. PV, KB, KAS, and TH performed all other experiments and data analysis. DK provided skin fibroblasts. MB and JNS helped with image analysis. All the authors reviewed and edited the manuscript.

## Supplementary Material

Supplemental data

Supporting data values

## Figures and Tables

**Table 2:**
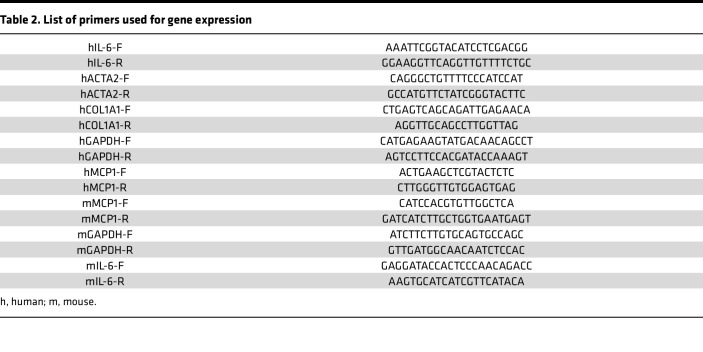
List of primers used for gene expression

**Table 1:**
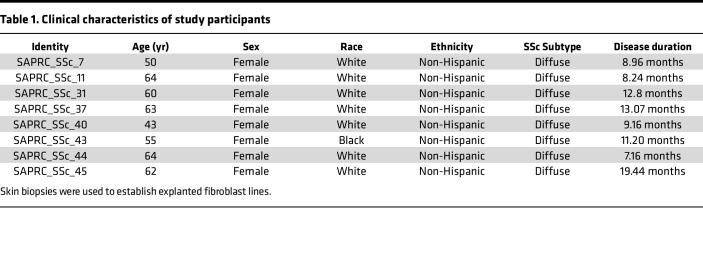
Clinical characteristics of study participants

## Version 1 10/17/2024

In-Press Preview

## Version 2 12/06/2024

Electronic publication
