# Supplementary material for: Inhibiting triggering receptor expressed on myeloid cells 1 signaling to ameliorate skin fibrosis
Source: JCI Insight. 2024 Dec 6;9(23):e176319. doi: 10.1172/jci.insight.176319 (PMC11623937; doi:10.1172/jci.insight.176319)
Supplement: Supplemental data [file jciinsight-9-176319-s159.pdf]

SUPPLEMENTARY FIGURE

Suppl. Figure 1

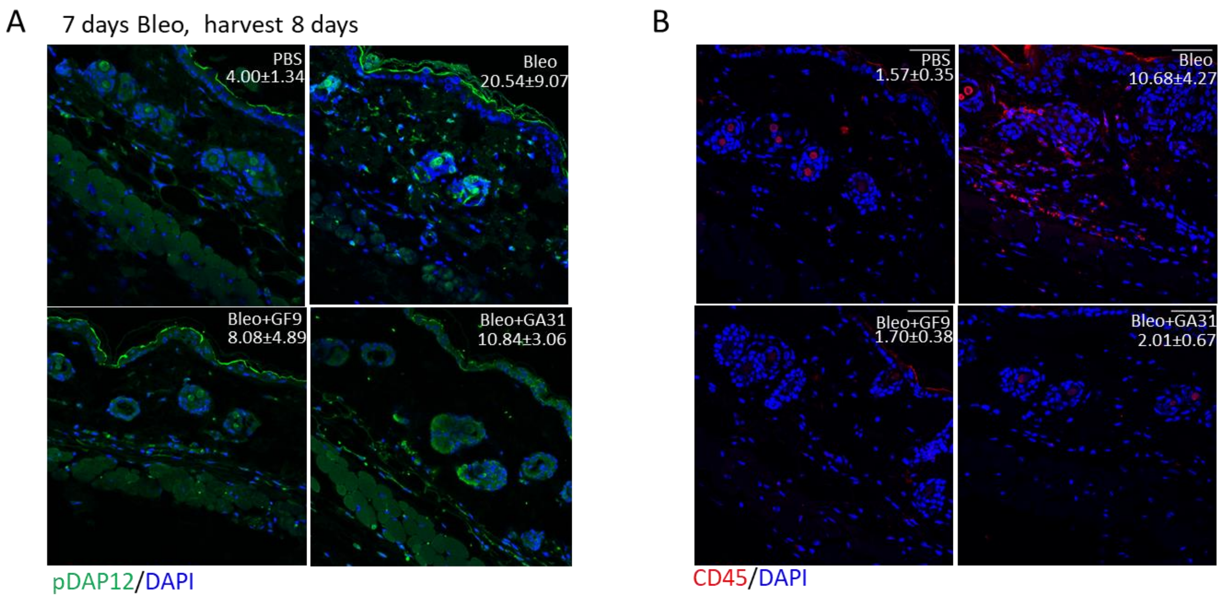

Supplementary Figure 1. **Inhibition of TREM-1 signaling by GF9 and GA31-LPC treatment attenuated pDAP12 and CD45 levels in mice skin tissue**. C57/BL6 mice received daily s.c. injections of PBS or bleomycin alone, or together with GF9 and GA31-LPC or vehicle. Mice were sacrificed on day 8. Skin tissues for the respective groups were immunolabelled with antibodies to (A) pDAP12 and (B) CD45 (bar, 50 µm). Quantitation of pDAP12 and CD45 positive cells (means ± SD; average from three randomly selected from four mice/group). Representative images.

Suppl. Figure 2

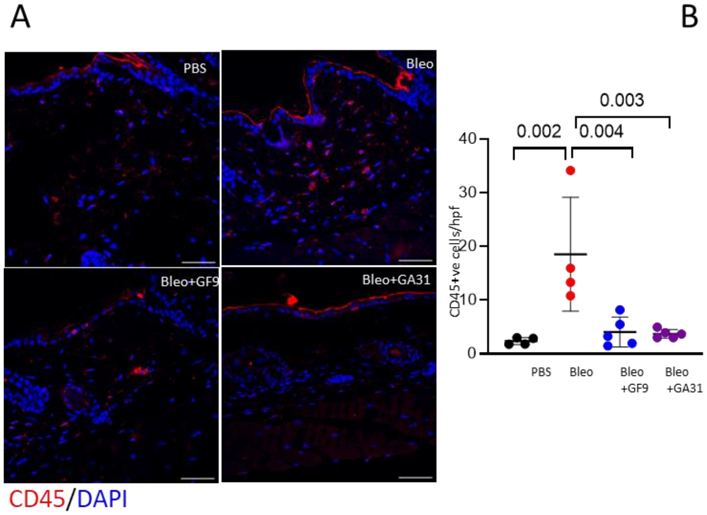

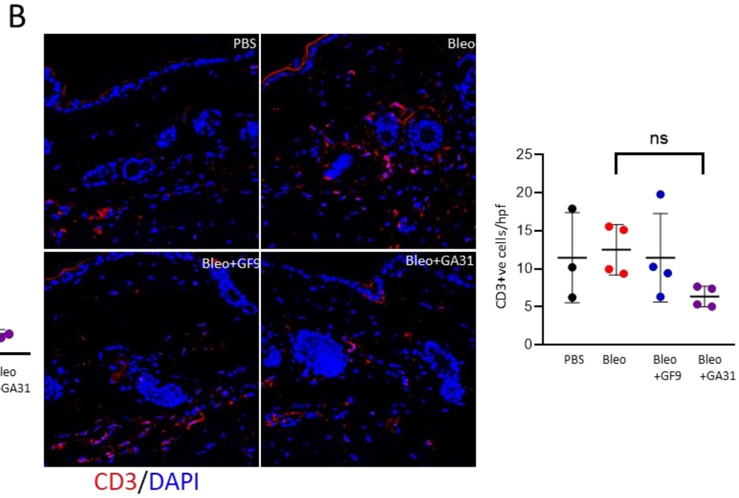

Supplementary Figure 2. GF9 and GA31-LPC concomitant treatment with bleomycin attenuated immune cell infiltrations. C57/BL6 mice received daily s.c. injections of PBS or bleomycin alone, or together with GF9 and GA31-LPC or vehicle. Mice were sacrificed on day 22. Skin tissues for the respective groups were immunolabelled with antibodies to A) CD45 for leukocytes and B) CD3 for T-lymphocytes (left panel, representative images, bar, 50µm) with quantification (right panel).

Suppl. Figure 3

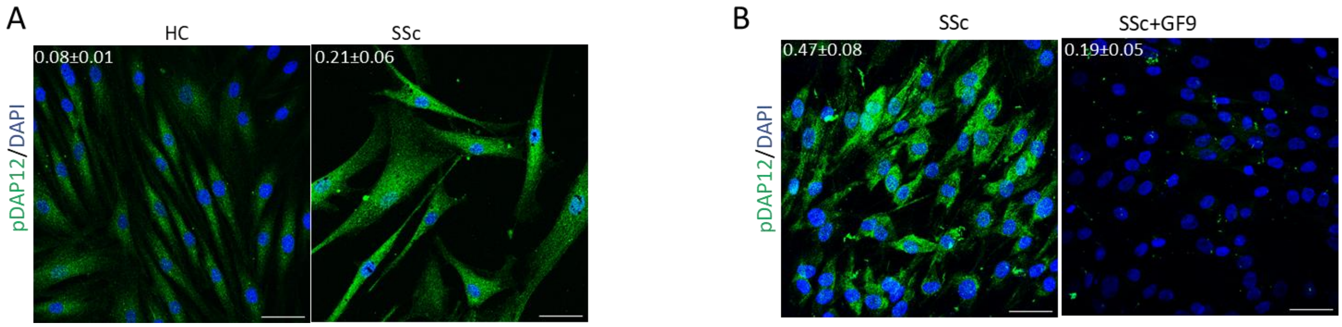

Supplementary Figure 3. TREM-1 downstream target is activated in SSc fibroblast. A. HC (n=2) and SSc fibroblasts (n=2) were immunolabeled using antibodies to pDAP12 (bar-50μm) and visualized by Nikon A1R laser scanning confocal microscope. Representative images. Relative fluorescence intensities (means ± SEM from 3 randomly selected regions). B. Confluent SSc skin fibroblasts (n=3, were incubated with GF9 for 24 h, and immunolabeled using antibodies to pDAP12 (bar-100μm). Representative images. Relative fluorescence intensities (means ± SEM from 3 randomly selected regions).

Suppl. Figure 4

10 days Bleo, harvest 21 days

A

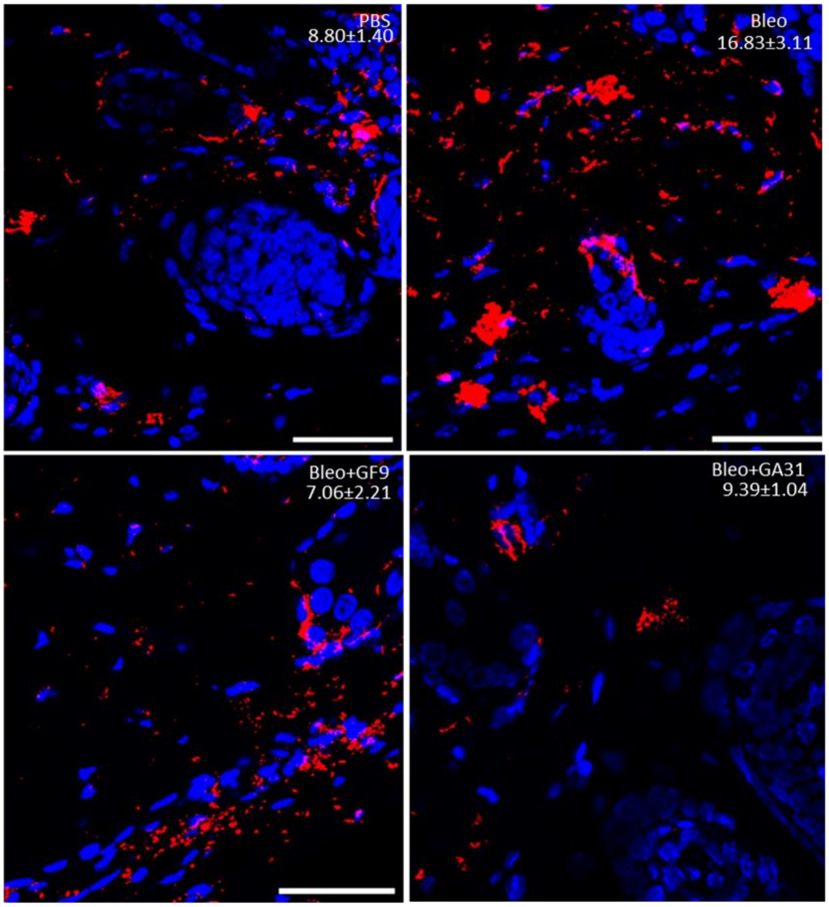

CD11b/DAPI

Supplementary Figure 4. **GF9 and GA31-LPC concomitant treatment with bleomycin attenuated myeloid cell infiltrations.** C57/BL6 mice received daily s.c. injections of PBS or bleomycin alone, or together with GF9 and GA31-LPC or vehicle. Mice were sacrificed on day 22. Skin tissues for the respective groups were immunolabelled with antibodies to A) CD11b for myeloid cells (representative images, bar, 50µm) with quantification (right panel). Quantitation of CD11b positive cells (means ± SEM; average from three randomly selected from four mice/group).
